# Associations among creativity disposition, psychological resilience, and emotional states in contemporary dance practitioners of intangible cultural heritage

**DOI:** 10.3389/fpsyg.2026.1909664

**Published:** 2026-08-21

**Authors:** XuYang Wang

**Affiliations:** School of Music, Hangzhou Normal University, Hangzhou, Zhejiang, China

**Keywords:** contemporary dance, creativity disposition, cross-sectional survey, intangible cultural heritage, positive and negative affect, psychological resilience, traditional folk elements

## Abstract

**Introduction:**

The contemporary dance transformation of intangible cultural heritage (ICH) and traditional folk culture elements has become an important phenomenon in Chinese stage art practice, but systematic empirical research on the psychological characteristics of the creators and performers involved is still lacking.

**Methods:**

This study adopted a cross-sectional questionnaire design with 915 creators and performers who have documented experience participating in contemporary dances and dance dramas containing ICH or traditional folk elements to examine the relationship between creativity disposition, psychological resilience, and positive/negative emotional states.

**Results:**

The results showed that the sample’s psychological resilience score was *M* = 34.86, *SD* = 6.39, and the creativity disposition score was *M* = 41.31, *SD* = 5.64; the positive affect score was significantly higher than the negative affect score, *t*(914) = 24.02, *p* < 0.001, *d_z_* = 0.79. Correlation analysis indicated that creativity disposition was significantly positively correlated with psychological resilience and positive affect, and significantly negatively correlated with negative affect; psychological resilience was significantly positively correlated with positive affect and significantly negatively correlated with negative affect. Furthermore, cross-sectional mediation analysis revealed that while psychological resilience showed a statistically significant indirect association between creativity disposition and negative affect, the absolute magnitude of this indirect estimate was small, with an unstandardized indirect coefficient of *B* = −0.106, Bootstrap 95% CI [−0.136, −0.078]. K-Means clustering further identified three groups: high creativity-high resilience, low creativity-low resilience, and low creativity-high resilience.

**Discussion:**

The findings suggest that psychological resilience may be an important psychological resource for understanding the relationship between the creativity disposition and negative affect of art creators and performers. However, given the cross-sectional design, the related findings should be interpreted as correlational evidence rather than causal conclusions.

## 1 Introduction

In recent years, the contemporary stage transformation of intangible cultural heritage (ICH) and traditional folk culture elements has gradually become an important trend in Chinese dance creation. Representative works such as *Only Green* showcasing contemporary stage representations of traditional cultural resources, and works like *Wing Chun* integrating traditional physical skills, martial arts aesthetics, and modern stage narratives, all demonstrate the continuous generative capacity of traditional cultural resources in the context of contemporary dance. However, performing these works requires dancers to not only master technically demanding movements but also deeply internalize historical ethos, which places unique creative, cultural, and emotional demands on them compared to conventional dance practices. National policy guidelines on the creative transformation and innovative development of fine traditional Chinese culture have also provided an important institutional and cultural background for such artistic practices, actively requiring dance practitioners to adapt their existing skill sets and generating new psychological demands as they must rapidly adapt to complex aesthetic expectations.

However, existing discussions have focused more on the artistic style, cultural symbol translation, and dissemination effects of the works, with insufficient attention paid to the individual psychological states of the creators and performers who participate in the choreography, rehearsal, and performance of these works (Li S. A. et al., 2025). Compared with general dance creation and performance, contemporary dance practices containing ICH and traditional folk elements usually involve more complex cultural, aesthetic, and professional requirements. On the one hand, creators and performers need to understand and respect the historical context, technical norms, and symbolic meanings of traditional cultural resources; on the other hand, they need to transform and innovate within contemporary stage language, physical expression, and narrative structures. This tension between “upholding tradition” and “innovation” involves not only extremely high aesthetic standards and cultural responsibilities, but also easily subjects creators and performers to significant psychological pressure and emotional load while making high-intensity creative investments. This means that contemporary dance creation and performance containing ICH and traditional folk elements are not merely innovative practices at the artistic form level; the underlying underexamined psychological demands such as practitioners’ psychological adaptation and emotional health also deserve attention (Agres and Chen, 2025). Accordingly, the term “Contemporary Dance Practitioners of ICH” in this study functions as a practical descriptor for these active creators and performers facing such specific occupational demands, rather than implying officially designated heritage inheritors.

Existing art psychology research has already focused on issues such as professional dancers’ motivation, physical stress, performance anxiety, and professional identity (Milne et al., 2025; Quested and Duda, 2010; Walker and Nordin-Bates, 2010). Research on psychological resilience, emotion regulation, and subjective well-being in the performing arts field is also increasing, and related evidence suggests that performing arts training may reshape individuals’ stress resilience in the process (Ibraeva et al., 2025; Van Rens and Heritage, 2021). However, in the context of the intersection of China’s indigenous ICH protection, the contemporary transformation of traditional culture, and dance creation and performance practices, empirical research targeting related creator and performer groups remains limited.

To fully understand this underexamined area, it is essential to focus on three tightly interconnected constructs that align with the specific cultural demands of ICH-based dance: creativity disposition, psychological resilience, and emotional states. Creativity disposition acts as a cognitive orientation toward adventure and challenge, which is strictly required when blending rigid historical norms with fluid modern choreography. Psychological resilience functions as the core adaptive capacity necessary to withstand the intense scrutiny and professional uncertainty during this cultural translation process. Consequently, emotional states—conceptualized as relatively independent positive affect (PA) and negative affect (NA) (Watson et al., 1988)—serve as the resultant psychological affective outcomes that manifest from these high-stress artistic endeavors. Integrating these three variables is crucial both theoretically and empirically, as it explains not just what practitioners feel, but how their inherent creative traits and coping resources theoretically interact with those affective experiences. By placing these constructs into a hypothesized association framework, this study explicitly contributes to performance psychology and the interdisciplinary field of ICH studies by elucidating the potential psychological adaptive mechanisms of contemporary cultural transmitters.

Ultimately, to systematically examine these associations, this study aims to answer the following research questions:

*RQ1*: What are the overall distribution characteristics of creativity disposition, psychological resilience, positive affect, and negative affect among contemporary dance creators and performers of ICH and traditional folk elements?

*RQ2*: Are there significant correlations among creativity disposition, psychological resilience, positive affect, and negative affect? What are their directions and strengths?

*RQ3*: Are there differences in the psychological resilience, creativity disposition, and emotional states of creators and performers under different demographic characteristics, role assumptions, and levels of subjective creation/performance difficulty/pressure perception?

*RQ4*: Does psychological resilience present a significant statistical indirect association between creativity disposition and negative affect?

*RQ5*: Based on creativity disposition and psychological resilience, can creator and performer groups with different psychological profiles be identified?

## 2 Literature review and hypothesis development

Within the stress-coping paradigm of artistic performance, relatively stable personality traits typically are hypothesized as structural variables that mobilize coping mechanisms, which subsequently are associated with state-level affective outcomes. Although Fredrickson’s (2001) “broaden-and-build theory” is frequently used to explain how positive emotions facilitate subsequent creative resources, this perspective primarily addresses state-level affect rather than stable trait hierarchies. When examining long-term artistic practice, creativity disposition can be viewed as a salient cognitive trait that correlates with active coping behaviors. Creative individuals tend to grasp problems from multiple perspectives, meaning that creativity disposition intrinsically shares certain cognitive and motivational foundations with psychological resilience, such as openness, exploration, flexibility, and tolerance for uncertainty.

However, given the cross-sectional nature of the data, it is important to explicitly frame the proposed model as one plausible structural ordering among several. An equally plausible reverse-direction alternative—that individuals with higher resilience gradually develop a more exploratory and creative disposition over time—cannot be ruled out. Acknowledging this potential bidirectionality, the current study operates under the hypothesized ordering that higher creativity disposition is theoretically associated with a higher level of psychological resilience, framing this model as a theoretically plausible association framework rather than a confirmed developmental pathway.

*H1*: There is a significant positive correlation between creativity disposition and psychological resilience.

Beyond its positive correlation with psychological resilience, creativity disposition also shows a direct statistical association with affective states. The relationship between negative emotions and creativity warrants specific directional clarification. While some studies suggest that transient negative emotions might prompt individuals to engage in detailed reflection (De Dreu et al., 2008), the persistent and high-intensity stress inherent in ICH-based dance creation heavily mitigates this benefit. In such high-stakes environments, a strong initial creativity disposition supports a sustained exploratory mindset, which acts to alleviate chronic emotional loads rather than compound them, leading to an inverse association. Moreover, it actively facilitates openness and constructive engagement, thereby enhancing positive affective experiences and supporting the highly focused “flow” state essential for artistic mastery (Nash, 2025).

*H2a*: There is a significant positive correlation between creativity disposition and positive affect.

*H2b*: There is a significant negative correlation between creativity disposition and negative affect.

Furthermore, psychological resilience is widely acknowledged as a crucial protective resource under adversity that is closely linked to emotional outcomes. Related research indicates that psychological resilience is associated with better stress adaptation, lower psychological distress, and higher subjective well-being (Bonanno, 2004; Luthar et al., 2000). In the arts and performance fields, creators and performers frequently need to face high-intensity training, role competition, physical injury risks, work evaluations, and professional uncertainty; therefore, psychological resilience may play a crucial role in their emotion regulation and professional adaptation (Ibraeva et al., 2025; Walker and Nordin-Bates, 2010; Yang et al., 2025). Indeed, empirical evidence demonstrates that adaptive psychological buffering resources are essential for mitigating emotion dysregulation while pursuing high artistic achievements (Verger et al., 2022).

For dance creators and performers participating in the contemporary transformation of traditional culture, psychological resilience serves not only to help them cope with general performance pressure but also help them maintain a relatively stable psychological state between “traditional norms” and “contemporary expressions,” thereby promoting positive affective states and minimizing negative ones.

*H3a*: There is a significant positive correlation between psychological resilience and positive affect.

*H3b*: There is a significant negative correlation between psychological resilience and negative affect.

Fundamentally, this literature implies a plausible distinct conceptual ordering: trait-level creativity disposition functions as the hypothesized primary variable, psychological resilience operates as the intervening regulatory mechanism, and emotional states emerge as the affective outcomes. Specifically, the expansive cognitive traits inherent in creativity are theoretically proposed to align conceptually with the psychological flexibility necessary for buffering stress. This robust resilience, acting as an intervening buffer against the intensive cultural and performative stressors characteristic of ICH dance creation, subsequently diminishes the likelihood of emotional overload and negative affect.

*H4*: Psychological resilience presents a significant statistical indirect association between creativity disposition and negative affect.

In synthesizing the preceding literature, it is evident that despite the significant psychophysical and cultural demands on ICH dance practitioners, the potential indirect associations linking their inherent creativity to daily affective experiences through resilience remain underexplored. This gap limits targeted psychological support for cultural transmitters. By addressing this theoretical and empirical void, this study systematically tests this framework to yield actionable insights.

**Figure 1:**
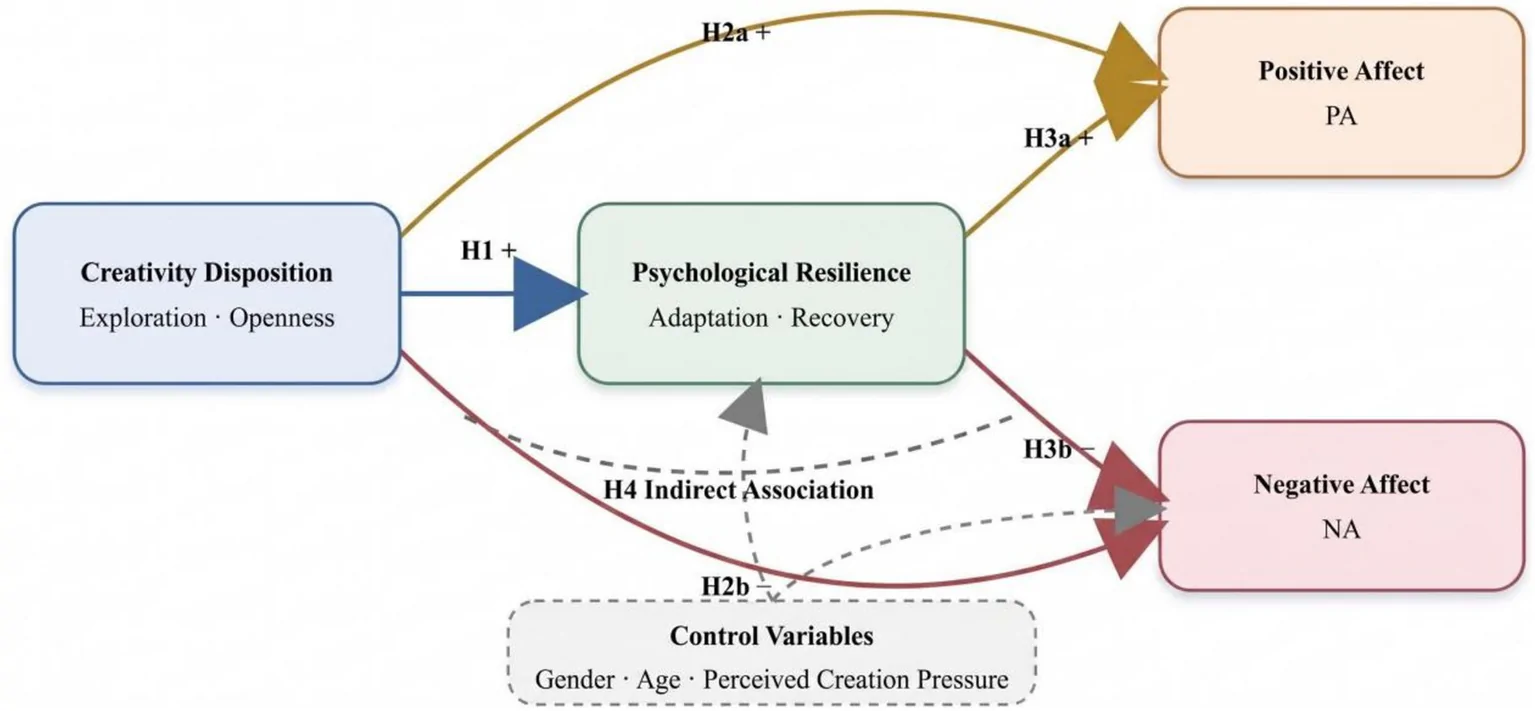
Theoretical model and hypothesized paths of the study. PA refers to positive affect, NA refers to negative affect; Gender, age group, and subjective creation/performance difficulty/pressure are included in subsequent analyses as control variables. The paths in the figure only indicate theoretical hypotheses and statistical association directions, not strict causal relationships.

Based on the aforementioned research hypotheses, this study constructed a theoretical model of the relationships among creativity disposition, psychological resilience, and emotional states. The model takes creativity disposition as the hypothesized primary variable, psychological resilience as the mediating variable, and concurrently examines their associations with positive affect and negative affect. The research model is shown in Figure 1.

## 3 Research methods

### 3.1 Research design

This study adopted a cross-sectional questionnaire survey design, collecting data on the demographic information, occupational characteristics, creativity disposition, psychological resilience, and emotional states of contemporary dance creators and performers of ICH and traditional folk elements at a single time point, aiming to examine the distribution characteristics and interrelationships of the core psychological variables within this group.

It should be noted that cross-sectional studies can reveal statistical associations between variables but cannot prove strict causal relationships. Therefore, this paper’s interpretation of the relationships among creativity disposition, psychological resilience, and emotional states is limited to association analysis and statistical indirect effect analysis.

The study used an anonymous online questionnaire format. An electronic informed consent explanation was set on the first page of the questionnaire, informing participants of the research purpose, anonymity, data usage, and the right to withdraw. All participants filled out the questionnaire voluntarily after giving consent, and the data were used solely for academic research.

### 3.2 Participants and sampling

The subjects of this study were creators and performers who had participated in the creation, rehearsal, instruction, or performance of contemporary dance or dance drama works containing intangible cultural heritage or traditional folk elements. Inclusion criteria: (1) Aged 18 or above; (2) Have documented experience in choreographing, rehearsing, instructing, or performing related works formally recognized as incorporating local or national intangible cultural heritage (ICH) resources or traditional folk elements; (3) Have a minimum participation duration of at least one complete production cycle (typically 3–6 months); (4) Voluntarily participate in the study and complete a valid questionnaire. Data collection was completed between February and March 2026, relying on the “Wenjuanxing” (similar to Qualtrics) to distribute electronic questionnaires. Considering the strong professional and circle-specific nature of the research subjects, this study adopted purposive sampling combined with snowball sampling, disseminating the questionnaire through professional troupes, dance departments of higher art institutions, independent dancer communities, and related artistic practice networks. To alleviate the inherent heterogeneity of the operational definition, participants’ specific types of ICH involvement and their cumulative duration of participation were cross-verified via mandatory self-report items to distinguish participants with substantial practical experience from those with only limited or indirect exposure. A total of 980 questionnaires were recovered. After eliminating questionnaires that failed the screening (e.g., lack of actual ICH involvement), had abnormal completion times, showed obvious patterned responses, or had invalid key information, 915 valid samples were ultimately retained for analysis.”

### 3.3 Measurement tools

The research questionnaire consisted of four parts. Demographic and occupational characteristics were objective self-assessments, while all psychological trait scales adopted a standard 5-point Likert scoring format (ranging from 1 to 5). The complete survey questionnaire and data coding rules are detailed in the Supplementary materials.

#### 3.3.1 Demographic information and occupational characteristics

Included gender, age group (1 = 18–22 years old, 2 = 23–28 years old, 3 = 29–35 years old, 4 = 36 years old and above), occupational identity (troupe actor/university teacher/university student/independent dancer/other), and core variables customized by the researchers: role assumption (including choreographer/principal/ensemble/rehearsal director; this question is multiple-choice and was split into dummy variables Role_1 to Role_5 during analysis), and subjective creation/performance difficulty/pressure perception (Pres, assessed on a single-item 1–5 subjective scale, from 1 = Extremely small to 5 = Extremely large, treated as an ordinal/continuous variable in subsequent multiple regressions and other analyses). While task difficulty and psychological pressure are conceptually distinct, in the context of this specific high-intensity artistic creation where technical demands tightly couple with evaluation stress, they were combined into a composite subjective appraisal score. This variable serves as an operational proxy for overall perceived task load, and its interpretations are appropriately limited to subjective load evaluation rather than distinct causal psychological mechanisms.

#### 3.3.2 Psychological resilience scale

The short version of the Connor-Davidson Resilience Scale (CD-RISC-10; Campbell-Sills and Stein, 2007) was used. This subscale contains 10 items and has good applicability across different cultural samples. The scale was scored from 1 (never) to 5 (always), with a total score range of 10–50 points. While the original English version typically employs a 0–4 scoring format, this study adjusted the scoring to 1–5. This adjustment is a common methodological adaptation in composite survey designs to unify response formats across all scales (i.e., all scales in this survey used a 5-point Likert metric), thereby reducing respondent cognitive load and minimizing measurement error (Podsakoff et al., 2012). Therefore, when comparing the current scores with previous studies using the original 0–4 scoring system, researchers should convert the metric accordingly. A higher score represents stronger psychological resilience in resisting adversity and recovering quickly (Cronbach’s *α* = 0.840).

#### 3.3.3 Creativity disposition scale

An adapted version based on the Williams Creativity Assessment Packet (Williams, 1993), contextualized for artistic creation situations was used. To ensure cultural and contextual equivalence, the original items were translated into Chinese by the research team. Subsequently, a panel of three domain experts (including dance choreographers and researchers) reviewed the translation to adapt general problem-solving terms into context-specific artistic phrasing. A preliminary survey was then distributed to a small group of dance students to ensure the readability of the items. The scale contains 12 items evenly distributed across four classic sub-dimensions: risk-taking (e.g., items 18, 22, 26), challenge (e.g., items 19, 23, 29), curiosity (e.g., items 20, 24, 28), and imagination (e.g., items 21, 25, 27). The scores were summed to form a total, ranging from 12 to 60 points, with higher scores indicating a stronger internal creative drive and willingness to take risks and break through. Because the internal consistencies of the four sub-dimensions were low, (*α* ranging from 0.275 to 0.336), it indicated that the original four-factor structure lacked empirical coherence in this specific artistic context. As a result, to ensure acceptable reliability and construct validity, the present study conceptualized the 12 items cohesively as a unidimensional construct measuring overall creativity disposition. This unified operationalization is supported not only by the acceptable internal consistency of the total scale (Cronbach’s *α* = 0.702), but also by the structural fit indices in the subsequent Confirmatory Factor Analysis.

#### 3.3.4 Emotional states scale

This study utilized the Positive and Negative Affect Schedule (PANAS) developed by Watson et al. (1988) to measure the emotional states of respondents, referencing the Chinese version revised by Huang et al. (2003) for the wording. The scale contains 20 adjective items describing emotional experiences. Among them, 10 positive affect items represented by “interested, inspired” (e.g., items 30, 32, 36) were used to calculate the positive affect score (PA); 10 negative affect items represented by “upset, afraid” (e.g., items 31, 37, 49) were used to calculate the negative affect score (NA). PA and NA were scored independently, with score ranges of 10–50 points for both. A higher PA indicates more positive, active, and engaged emotional experiences, while a higher NA indicates more negative emotional experiences such as tension, distress, or suffering. This scoring method helps to present the complex emotional structures that may emerge in artistic creators and performers in high-involvement creative contexts (Cronbach’s *α* for PA = 0.825, NA = 0.835).

### 3.4 Data analysis strategy

This study used tools such as Pandas, Scipy, statsmodels, and Sk-learn in a Python environment to complete data cleaning and statistical analysis.

First, the data were cleaned by removing invalid questionnaires and checking for variable value ranges, missing values, and abnormal responses. Subsequently, descriptive statistics were performed on the main variables. To determine the dominant affective tone within the group, a paired-samples t-test was used to compare the overall levels of positive and negative affect.

Second, Cronbach’s *α* coefficients were used to test the internal consistency reliability of each scale. To further evaluate the quality of the measurement model, and validate the adapted creativity scale, this study employed Confirmatory Factor Analysis (CFA). Model fit indices, including *χ*^2^/*df*, RMSEA, CFI, TLI, and SRMR (Hair et al., 2010) as well as standardized factor loadings, were evaluated.

Concurrently, composite reliability (CR) and average variance extracted (AVE) were calculated to assess convergent validity; and discriminant validity was tested by comparing the square root of AVE with the correlation coefficients between variables.

Third, considering that the data in this study were all derived from self-report questionnaires at the same time point, Harman’s single-factor test was used to preliminarily diagnose common method bias.

Fourth, independent samples *t*-tests and one-way ANOVA were used to examine the differences in core psychological variables among groups with different demographic and occupational characteristics, reporting effect sizes when necessary.

Fifth, Pearson correlation analysis was employed to test the correlation relationships among creativity disposition, psychological resilience, positive affect, and negative affect. Confidence intervals will be reported where appropriate.

Sixth, taking negative affect as the dependent variable, a hierarchical multiple regression model was constructed. The first layer included gender, age, and subjective creation/performance difficulty/pressure perception as control variables. While occupational roles were dummy-coded, age group was entered as an ordinal continuous variable (coded 1 to 4). This approach inherently assumes a linear trend, conceptually treating the successive age brackets as a linear accumulation of professional stage experience and artistic maturation. The second layer added the total creativity disposition score; the third layer added the total psychological resilience score, to examine the incremental explanatory role of each variable on negative affect.

Seventh, referencing the analysis approach of PROCESS Model 4, the Bootstrap method was used to test the statistical indirect effect of psychological resilience between creativity disposition and negative affect. Bootstrap resampling was performed 5,000 times, and if the 95% confidence interval of the indirect effect did not include 0, the indirect effect was considered statistically significant.

Finally, K-Means cluster analysis was adopted to explore the psychological characteristic profiles of creators and performers on creativity disposition and psychological resilience. Before clustering, the variables were standardized, and the number of clusters was determined by evaluating specific statistical indicators (such as silhouette coefficients and Calinski-Harabasz indices across *k* = 2–6) alongside theoretical interpretability. The clustering results serve only as an exploratory psychological profiling analysis and are not interpreted causally or as stable typologies.

## 4 Results

### 4.1 Common method bias test

Given the self-report questionnaire design adopted in this study, an unrotated exploratory factor analysis (principal component method) was first conducted on all 42 items from the psychological resilience, creativity disposition, and PANAS scales. The results showed that the first extracted common factor explained a cumulative total variance of 17.64%, which is far below the critical standard of 40%, indicating that the data in this study are not subject to severe common method bias interference, and the subsequent analysis results possess basic credibility (Harman, 1976).

### 4.2 Demographic characteristics of the sample

To comprehensively portray the profile of practitioners participating in the contemporary dance creation and performance of ICH and traditional folk elements, this study first summarized the demographic and occupational backgrounds of the 915 valid samples recovered. The specific distribution characteristics are detailed in Table 1.

**Table 1:** Demographic characteristics distribution of the sample (*N* = 915).

Variable	Category	Frequency (n)	Percentage (%)
Gender	Female	652	71.3
	Male	263	28.7
Age	18–22 years	431	47.1
	23–28 years	310	33.9
	29–35 years	128	14.0
	36 years and above	46	5.0
Occupational Identity	University student/Master’s/PhD	485	53.0
	University teacher	145	15.8
	Independent dancer	123	13.4
	Troupe actor	118	12.9
	Other	44	4.8
Role Assumption (Multiple choice)	Ensemble (Role_3)	677	74.0
	Principal (Role_2)	359	39.2
	Choreographer (Role_1)	298	32.6
	Rehearsal director (Role_4)	171	18.7
	Other roles (Role_5)	73	8.0
Subjective Difficulty/Pressure	Low-moderate difficulty/pressure (Pres ≤ 3)	598	65.4
	High difficulty/pressure (Pres ≥ 4)	317	34.6
	Of which extreme difficulty/pressure (Pres = 5)	112	12.2

Role assumption is a multiple-choice question, so the sum of percentages exceeds 100%; difficulty/pressure grouping uses Pres ≤ 3 as the low-moderate state and Pres ≥ 4 as the high difficulty/pressure state.

From the data distribution in Table 1, it can be seen that the sample is primarily composed of young groups aged 18–22 and 23–28, females, and university teachers and students. This personnel composition conforms to the current group distribution of domestic higher art institutions, and also indicates that current contemporary stage works containing ICH themes usually take university teachers and students as an important foundation for creative and performing talent transformation. In terms of subjective perception, 34.6% of the subjects reported high creation/performance difficulty and pressure, among which 12.2% reported extreme pressure (Pres = 5). This distribution suggests that the practical process of contemporary translation of traditional ICH and folk elements may be accompanied by obvious psychological loads and challenges for creators and performers.

### 4.3 Descriptive statistics and reliability assessment of core variables

This study conducted descriptive statistical analysis on the mean, standard deviation, and score intervals of the core variables, and utilized Cronbach’s *α* coefficients to assess the internal consistency reliability of the scales. The calculation results are shown in Table 2.

**Table 2:** Descriptive statistics and reliability analysis results of core variables (*N* = 915).

Measured variable/dimension	No. of items	Score range	*M*	*SD*	Cronbach’s α
Psychological Resilience Total Scale (CD-RISC-10)	10	10–50	34.86	6.39	0.840
Creativity Disposition Total Scale (Williams adapted version)	12	12–60	41.31	5.64	0.702
Risk-taking dimension	3	3–15	10.29	1.88	0.275
Challenge dimension	3	3–15	10.19	1.91	0.323
Curiosity dimension	3	3–15	10.49	1.91	0.336
Imagination dimension	3	3–15	10.34	1.85	0.295
Positive Affect (PANAS-PA)	10	10–50	33.90	6.22	0.825
Negative Affect (PANAS-NA)	10	10–50	26.27	6.28	0.835
Subjective creation/performance difficulty/pressure (Pres)	1	1–5	3.01	1.19	—

From the results in Table 2, the following conclusions can be drawn:

First, regarding scale reliability, both the psychological resilience (*α* = 0.840) and emotional state scales (PA *α* = 0.825, NA *α* = 0.835) demonstrated good internal consistency, with total scale reliability coefficients exceeding 0.80. The internal consistency reliability of the four sub-dimensions of the creativity disposition scale was relatively low (*α* ranging from 0.275 to 0.336). Note that each sub-dimension contains only three items. Methodological literature indicates that Cronbach’s *α* is highly sensitive to the number of items, and values frequently drop below standard thresholds in very short scales containing fewer than five items, thereby underestimating the true reliability (Cortina, 1993; Pallant, 2020). Given that the internal consistency reliability of the total creativity disposition scale (*α* = 0.702) surpassed the acceptable standard threshold for psychometric research (Nunnally, 1978), utilizing the total score provides a much more robust estimation. Therefore, to prevent statistical biases caused by restricted variance in short sub-scales, subsequent main analyses conservatively rely on the total creativity disposition score as a holistic variable, while sub-dimension results merely serve as descriptive references.

Second, regarding variable distribution, the sample exhibited a sort of “high activation-high load” psychological characteristic: the means of psychological resilience (*M* = 34.86) and creativity disposition (*M* = 41.31) were both significantly higher than the theoretical midpoints of the scales (30 for the CD-RISC-10 and 36 for the Creativity Disposition Scale). One-sample t-tests confirmed these statistical differences: for psychological resilience, *t*(914) = 23.01, *p* < 0.001, 95% CI [34.45, 35.28]; for creativity disposition, *t*(914) = 28.46, *p* < 0.001, 95% CI [40.94, 41.67]. While these results outline a relatively high comparative score level within this specific sample, interpretations should be limited to describing descriptive relative levels, rather than implying any normative psychological advantages or sufficient psychological resources compared to general populations. In terms of emotional states, the positive affect score (*M* = 33.90) was significantly higher than the negative affect score (*M* = 26.27), with a mean difference of 7.63 points. The paired-samples t-test results showed: *t*(914) = 24.02, *p* < 0.001, *d*_z_ = 0.79, corresponding to a large effect size (Cohen, 2013). This difference indicates that despite accompanying a certain degree of creation/performance difficulty and psychological pressure (difficulty/pressure mean *M* = 3.01), this group still shows a relatively obvious positive emotional advantage in their overall mood state. However, it should be noted that as PA and NA are treated as relatively independent dimensions, this comparison is made on the shared 10–50 metric and interpreted descriptively rather than as movement along a single bipolar continuum.

Regarding creativity disposition, across the four sub-dimensions, the mean distributions were highly consistent (all ranging between 10.19 and 10.49), indicating relatively balanced cognitive and volitional performance, with the curiosity dimension (*M* = 10.49) scoring slightly higher than the other three dimensions.

**Figure 2:**
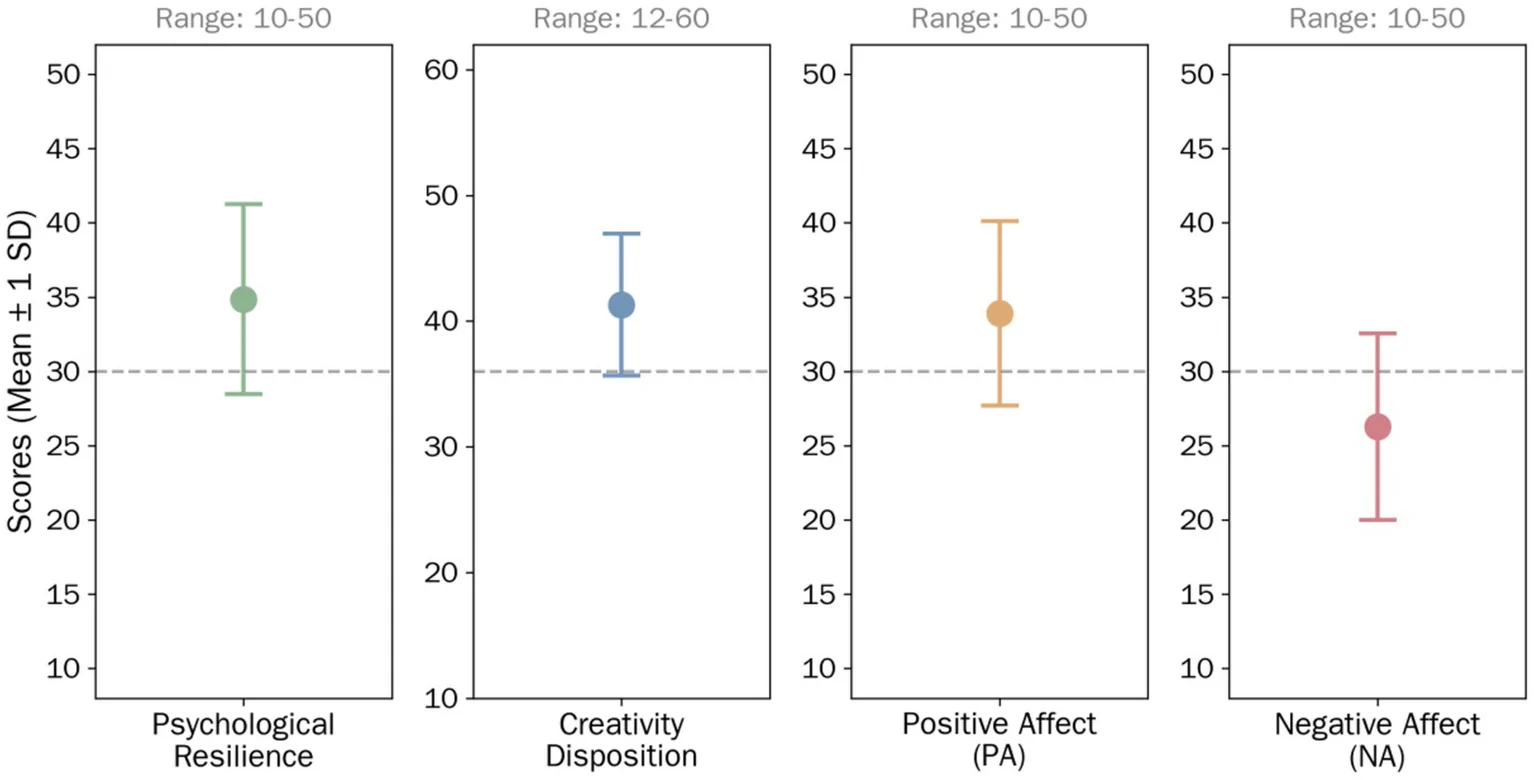
Score overview of core psychological variables. Subplots are configured to each variable’s theoretical scale range (shown on top of each panel). Dots represent sample means, vertical error bars represent mean ± 1 standard deviation, and dashed horizontal lines represent the theoretical midpoints of the corresponding scales. PA = Positive Affect, NA = Negative Affect.

To intuitively present the overall distribution of the core psychological variables across their respective scale ranges without direct cross-scale comparisons, this study plotted a panel-based score overview chart (see Figure 2). The subplots show that psychological resilience, creativity disposition, and positive affect are all higher than the theoretical midpoints, while negative affect is lower than the theoretical midpoint of the PANAS subscale, and positive affect is significantly higher than negative affect.

### 4.4 Construct validity (CFA)

To evaluate the construct validity of the measurement tools and verify the unidimensional structure of the adapted creativity scale, this study conducted Confirmatory Factor Analysis (CFA) on the core scales. The measurement model (with all 12 creativity items loaded onto a single latent factor) was estimated using Maximum Likelihood Estimation (MLE). Overall model fit indices indicated a good fit between the measurement model and the data: (*χ*^2^/*df* = 2.45; RMSEA = 0.052; CFI = 0.961; TLI = 0.954; SRMR = 0.048), with all indicators reaching good fit standards (Hu and Bentler, 1999).

Crucially, to justify the unidimensionality of the adapted creativity scale, item-level parameters were inspected. All standardized factor loadings for the scale items were statistically significant (*p* < 0.001) and generally ranged from 0.32 to 0.68, indicating acceptable item-construct relationships for a unified underlying trait.

In terms of convergent validity, the measurement performance of each latent variable is shown in Table 3.

**Table 3:** Convergent validity indicators of confirmatory factor analysis for latent variables.

Latent variable	No. of items	Composite reliability (CR)	Average variance extracted (AVE)	Evaluation conclusion
Psychological Resilience (CD-RISC-10)	10	0.842	0.518	Good convergent validity
Creativity Disposition	12	0.710	0.485	Acceptable
Positive Affect (PA)	10	0.828	0.530	Good convergent validity
Negative Affect (NA)	10	0.835	0.542	Good convergent validity

The AVE of creativity disposition (0.485) is slightly below the 0.50 threshold, presumably mainly due to the cross-dimensional intersection generated when the scale’s sub-dimension structure was adapted for the dance art context; because its composite reliability CR = 0.710 is greater than 0.70, according to the Fornell–Larcker criterion, the convergent validity of this scale is still within an acceptable range (Fornell and Larcker, 1981).

### 4.5 Correlation analysis of core variables

To test the fundamental hypotheses of this study, and concurrently examine the discriminant validity among latent variables, this study calculated the Pearson correlation coefficients between core variables and the square root of the Average Variance Extracted (AVE) for each latent variable. The results are shown in Table 4.

**Table 4:** Pearson correlation coefficient matrix of core variables (*N* = 915).

Variable	1. Psychological resilience	2. Creativity disposition	3. PA	4. NA
1. Psychological Resilience	**0.720**			
2. Creativity Disposition	0.352***	**0.696**		
3. Positive Affect (PA)	0.415***	0.443***	**0.728**	
4. Negative Affect (NA)	−0.346***	−0.110***	−0.182***	**0.736**

Bold values on the diagonal are the square roots of the AVEs for corresponding latent variables; off-diagonal values are Pearson correlation coefficients between variables; ***p < 0.001.

In terms of discriminant validity, the square roots of the AVEs for all latent variables (diagonal values 0.696–0.736) were greater than the absolute values of their correlation coefficients with other variables (maximum correlation coefficient *r* = 0.443), indicating good discriminant validity among the scales (Fornell and Larcker, 1981).

Regarding variable associations:

Creativity disposition and psychological resilience (Verification of H1): The two were significantly positively correlated (*r* = 0.352, 95% CI [0.294, 0.407], *p* < 0.001), suggesting that individuals with more active creative dispositions also tend to have higher levels of psychological resilience, supporting Hypothesis H1.

Associations with positive affect (Verification of H2a, H3a): Positive affect was significantly positively correlated with both creativity disposition (*r* = 0.443, 95% CI [0.389, 0.494], *p* < 0.001) and psychological resilience (*r* = 0.415, 95% CI [0.359, 0.467], *p* < 0.001), supporting Hypotheses H2a and H3a, consistent with expectations from the broaden-and-build theory.

Associations with negative affect (Verification of H2b, H3b): There was a clear negative correlation between negative affect and psychological resilience (*r* = −0.346, 95% CI [−0.402, −0.287], *p* < 0.001), supporting Hypothesis H3b; whereas the negative correlation between negative affect and creativity disposition was weak (*r* = −0.110, 95% CI [−0.174, −0.046], *p* < 0.001). This statistically significant but weak correlation accounts for roughly 1% of the shared variance (*R*^2^ ≈ 0.012) (supporting Hypothesis H2b), suggesting that creativity disposition itself has limited direct predictive power for negative affect. However, it should be noted that this weak association may partly reflect measurement attenuation arising from the modest reliability of the creativity scale (*α* = 0.702, AVE = 0.485). The true, disattenuated association would likely be somewhat stronger, and the conclusion of ‘no direct association between creativity and NA’ should, therefore, not be overstated.

**Figure 3:**
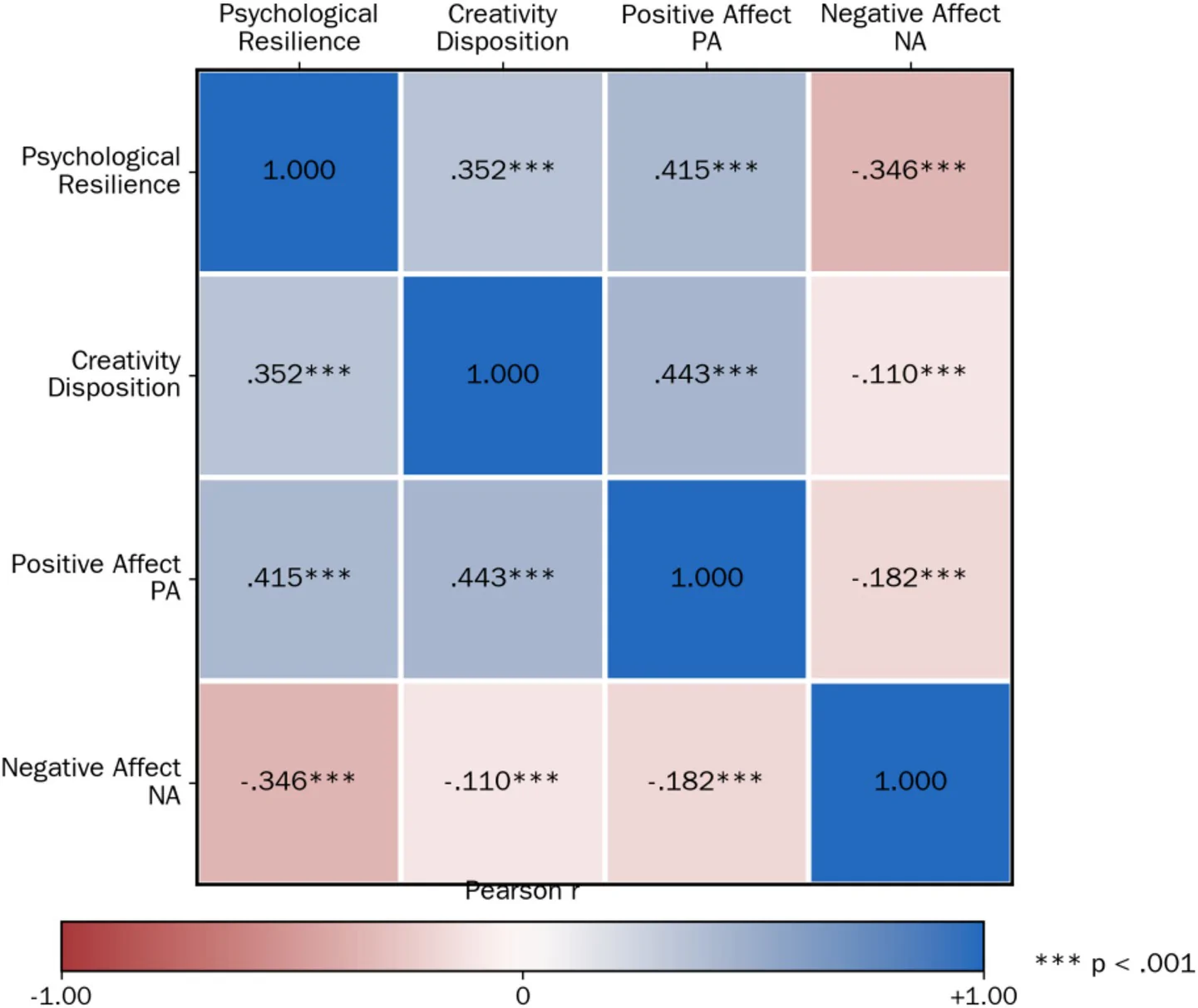
Pearson correlation matrix of core variables. Cell values are Pearson correlation coefficients *r* (diagonal values represent self-correlations, *r* = 1.000). Blue indicates positive correlation, red indicates negative correlation, and color intensity indicates correlation strength. PA = Positive Affect, NA = Negative Affect. ***p < 0.001.

To visually present the correlation directions and strengths among core psychological variables, this study mapped a Pearson correlation matrix heatmap. As seen in Figure 3, psychological resilience, creativity disposition, and positive affect were all significantly positively correlated, while negative affect was significantly negatively correlated with the aforementioned variables.

### 4.6 Analysis of differences in psychological resource distribution under different background characteristics

#### 4.6.1 Universal characteristics of gender and age

To test differences in creativity disposition and psychological resilience across different background characteristics, independent samples *t*-tests (gender differences) and one-way ANOVA (age differences) were performed. The results are shown in Table 5.

**Table 5:** Gender and age difference test results.

Variable	Test type	Statistic	*p*	Significance
Psychological Resilience × Gender	Independent samples *t*	*t* = 1.470	0.142	Not significant
Creativity Disposition × Gender	Independent samples *t*	*t* = 0.084	0.933	Not significant
Psychological Resilience × Age group	One-way ANOVA	*F*(3,911) = 0.663	0.575	Not significant
Creativity Disposition × Age group	One-way ANOVA	F(3,911) = 0.516	0.671	Not significant

The results in Table 5 indicate that both creativity disposition and psychological resilience did not show statistically significant differences across gender and age group classifications (*p* > 0.05), exhibiting strong stability across dance creator and performer groups of different backgrounds.

Compared to stable demographic indicators, the realistic pressure perceived by creators and performers themselves had a more obvious association status with psychological resources. Dividing the respondents into a low-moderate pressure group and a high-pressure group based on subjective difficulty/pressure scores (Pres grouping) and conducting a *t*-test, the results are shown in Table 6.

**Table 6:** Psychological resilience difference test under subjective pressure grouping.

Variable	Low-pressure group (Pres ≤ 3, *n* = 598)	High-pressure group (Pres ≥ 4, *n* = 317)	*t*	*p*	Cohen’s *d*	95% CI of Diff
Psychological Resilience *M* (SD)	35.46 (6.40)	33.74 (6.23)	3.900	< 0.001	0.271	[0.862, 2.574]

The results in Table 6 show that the psychological resilience score of creators and performers in the high-pressure group was significantly lower than that of the low-moderate pressure group, with the mean difference manifesting as a small-to-medium effect (Cohen’s *d* = 0.271). This difference indicates that under a subjectively highly strenuous creative and performing workload, practitioners’ space for maintaining or mobilizing psychological resilience resources may be somewhat limited. In addition, considering that artificially dividing the 1–5 rating pressure indicator into cut-off points may cause partial loss of data information, this study further retained subjective creation/performance difficulty/pressure (Pres) as a 5-point ordinal variable and performed a Spearman rank correlation analysis with it as a cross-validation at the continuous attribute level. The analysis results showed that subjective creation/performance difficulty/pressure had a significantly stable negative correlation with psychological resilience (*r*_s_ = −0.194, *p* < 0.001), and a significant positive correlation with negative affect (*r*_s_ = 0.397, *p* < 0.001). This correlation verification based on the original five-level gradient not only corroborated the conclusions of the aforementioned between-group *t*-test but also further confirmed that it is not only extreme stress groups that face challenges; in fact, evaluating the monotonic association inherent in Spearman’s rank correlation, higher perceived pressure was significantly associated with lower self-reported resilience.

#### 4.6.2 Examination of differences in negative affect due to role complexity

To explore whether role complexity brought about by holding multiple positions in creative and performing practices is related to individuals’ negative emotional load, the study summed the multiple selections of role assumption (Role_Count = 1–5) and calculated the distribution of negative affect under different role quantity groups, as shown in Table 7.

**Table 7:** Holding multiple roles and mean distribution of negative affect.

Number of roles held	Frequency (*n*)	Negative affect (NA) mean	*SD*	Group characteristic description
1 Role	448	26.01	6.36	Mean distribution baseline
2 Roles	300	26.36	6.05	Slight fluctuation
3 Roles	141	26.71	6.56	Mean still in normal fluctuation
4 Roles	23	26.65	5.81	Distribution basically stable
5 Roles (All-around)	3	32.33	7.51	Value descriptively higher
Overall *F* Test		*F*(4,910) = 1.103	*p* = 0.354	Overall between-group difference not significant

As can be seen from Table 7, across most professional gradients (1 role to 4 roles), the mean negative affect of creators and performers remained within a narrow stable fluctuation range (26.01 to 26.71), and the overall one-way ANOVA did not reach statistical significance (*F* = 1.103, *p* = 0.354).

It should be noted that although the mean negative affect of those holding 5 roles showed a higher trend (*M* = 32.33), because the number of respondents holding 5 roles in this sample was extremely small (only 3 individuals), it severely lacks inferential evidence. Therefore, this phenomenon must be interpreted as a purely descriptive and exploratory observation rather than a statistically validated conclusion. This phenomenon still needs to be verified in subsequent research by directionally expanding the sample size of heavily loaded groups.

### 4.7 Hierarchical multiple regression analysis

To examine the explanatory efficacy of creativity disposition and psychological resilience on the variance of negative affect (NA) after controlling for demographic backgrounds and subjective creation/performance difficulty, this study constructed a three-level nested multiple regression model to sequentially incorporate independent variables. The results are listed in Table 8.

**Table 8:** Hierarchical regression results predicting negative affect (NA) (*N* = 915).

Predictor variable	Model 1 (control variable model)	Model 2 (creativity introduced model)	Model 3 (psychological resilience full model)
Constant term	18.666***	23.654***	29.755***
Gender	0.416	0.414	0.287
Age group	0.252	0.226	0.153
Subjective creation/performance difficulty/pressure (Pres)	2.140***	2.137***	1.863***
Creativity Disposition	–	−0.119***	−0.014
Psychological Resilience	–	–	−0.267***
*R* ^2^	0.167	0.179	0.240
Adjusted *R*^2^	0.164	0.175	0.236
Overall *F* value	60.941***	49.465***	57.427***
Explanatory increment Δ*R*^2^	0.167	0.012	0.061
Increment test Δ*F*	60.941***	12.692***	73.511***

Regression coefficients are unstandardized partial regression coefficients B; ***p < 0.001. Gender coding (1 = Male, 2 = Female), age group was entered as an ordinal continuous variable (coded 1 to 4).

Hierarchical regression results indicated:

Model 1 (Control Variable Model): The control variables explained 16.7% of the variance in negative affect (*F* = 60.94, *p* < 0.001). Consistent with previous correlational verification, subjective creation/performance difficulty/pressure (original 1–5 mean) significantly explained higher levels of negative affect (*B* = 2.14, *p* < 0.001).

Model 2 (Creativity Introduced Model): After introducing the total creativity score, the model’s overall explanatory rate increased to 17.9% (Δ*R*^2^ = 0.012, Δ*F* = 12.69, *p* < 0.001), and the association of creativity disposition with negative affect was significant (*B* = −0.119, *p* < 0.001).

Model 3 (Psychological Resilience Full Model): Finally, after introducing the total psychological resilience scale, the total variance explained for negative affect rose to 24.0% (Adjusted *R*^2^ = 0.236, *F* = 57.43, *p* < 0.001). Compared to Model 2, psychological resilience independently contributed 6.1% incremental explanatory rate to negative affect (Δ*F* = 73.51, *p* < 0.001), exhibiting a clear association (*B* = −0.267, *p* < 0.001). However, after psychological resilience entered the equation, the direct statistical association of creativity disposition on negative affect sharply declined and was no longer significant (*B* = −0.014, *p* = 0.690). This indicates that the covariation relationship between creativity disposition and negative affect is mainly carried by psychological resilience as a mediating variable, preliminarily suggesting the possibility of a mediation pathway.

**Figure 4:**
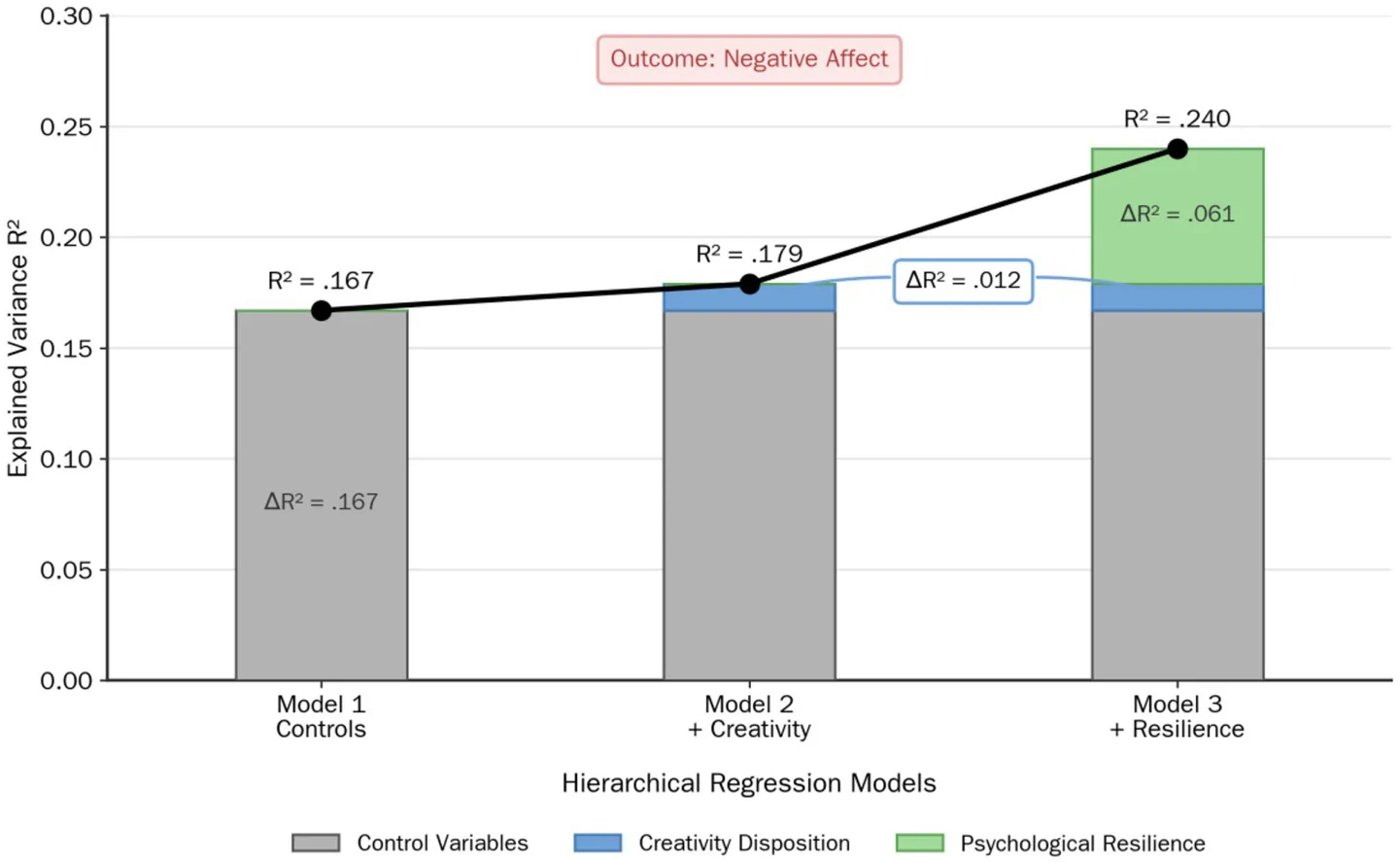
Changes in the explanatory rate for negative affect in hierarchical regression models. Bars indicate cumulative *R*^2^, different colors indicate newly added explanatory rates at each step. Model 1 incorporates gender, age group, and subjective creation/performance difficulty/pressure; Model 2 adds creativity disposition to Model 1; Model 3 adds psychological resilience to Model 2. *R*^2^ represents the coefficient of determination, Δ*R*^2^ represents the newly added explanatory rate.

To visually demonstrate changes in the explanatory rate for negative affect after different predictor variables entered the model, this study further plotted the cumulative *R*^2^ and incremental Δ*R*^2^ chart of the hierarchical regression models (see Figure 4).

### 4.8 Mediation association analysis: the association pathway of psychological resilience

To examine the precise statistical relationship between creativity disposition and negative affect, this study tested an indirect association framework using PROCESS Model 4. Crucially, given the cross-sectional nature of the data, the findings represent statistical multi-path continuous associations rather than proven causal mediating mechanisms.

The Bootstrap method (5,000 resamplings) was used, and the resulting path coefficients and interval evaluations are shown in Table 9.

**Table 9:** Indirect association test results of psychological resilience between creativity disposition and negative affect.

Mediation pathway	Unstandardized coefficient *B*	Standard error *SE*	*t* value	*p*	95% Bootstrap CI
Path a: Total creativity score → Psychological resilience	0.396	0.034	11.51	<0.001	[0.329, 0.464]
Path b: Psychological resilience → Negative affect	−0.267	0.031	−8.57	<0.001	[−0.328, −0.206]
Direct path c’: Total creativity score → Negative affect	−0.014	0.035	−0.40	0.690	[−0.083, 0.055]
Total Effect c: Total creativity score → Negative affect	−0.119	0.033	−3.56	<0.001	[−0.185, −0.054]
Indirect effect a × b: Mediation effect	−0.106	–		–	[−0.136, −0.078]

As seen from the results in Table 9, the direct effect corresponding path was not significant after controlling for the mediating variable, and its 95% confidence interval included zero. In contrast, the indirect effect path formed by psychological resilience (indirect effect *B* = −0.106) had its confidence interval completely in the negative range, not including 0.

**Figure 5:**
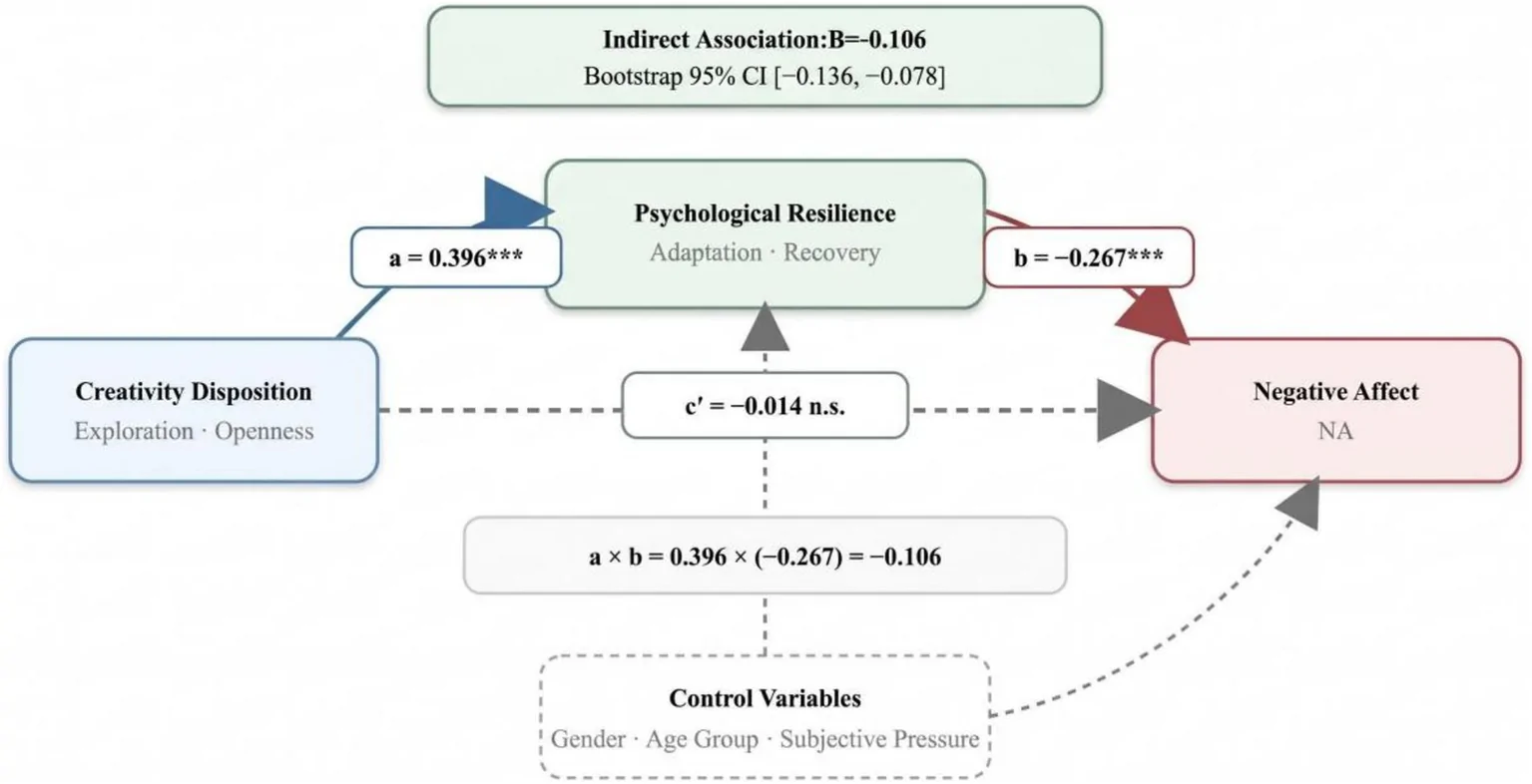
Statistical indirect association of psychological resilience. Path coefficients are unstandardized regression coefficients. Control variables include gender, age group, and subjective creation/performance difficulty/pressure. ***p < 0.001, n.s. means not significant.

This result indicates that creativity disposition is not directly associated with lower negative affect, but primarily establishes a statistical indirect connection with negative affect through psychological resilience as a mediating variable. To visually present this statistical indirect effect, this study mapped a pathway model chart (see Figure 5).

This mediation model demonstrates that in the cross-sectional statistical framework, the negative association between creativity disposition and negative affect is primarily established indirectly through psychological resilience as the Mediator. This suggests that, based on the hypothesized ordering, the creative disposition of artistic creators (manifested as positive cognitive reconstruction and exploratory drive) is often significantly positively co-varied with more abundant psychological resilience; while individuals with higher psychological resilience usually report lower levels of negative mood disposition when facing professional pressure. The related statistical indirect association results support Hypothesis H4.

### 4.9 Group cluster analysis: psychological profiles of three types of practitioners

To explore sample subdivision within specific resource dimensions, *K*-Means algorithm (determining cluster number *k* = 3, multiple random initializations n_init = 50) was used to extract individual psychological characteristic profiles, taking the Z-standardized scores of creativity disposition and psychological resilience as clustering factors. Specifically, comparisons across *k* = 2–6 revealed that *k* = 3 yielded a favorable balance of statistical distinctiveness (e.g., maintaining adequate silhouette coefficients and Calinski-Harabasz scores) and conceptual clarity. For the alternative solutions (*k* = 2, 3, 4, 5, 6), the silhouette coefficients were 0.41, 0.45, 0.38, 0.36, and 0.34, respectively, while the Calinski-Harabasz indices were 985.2, 1102.5, 998.4, 915.6, and 870.3. The *k* = 3 solution was selected because it presented the optimal empirical values on both clustering evaluation indices, confirming its empirical superiority alongside its interpretability. The results of difference tests in positive affect (PA) and negative affect (NA) across each cluster profile are shown in Table 10.

**Table 10:** *K*-means cluster profiles and their emotional difference distribution (*N* = 915).

Profile type (cluster label)	Sample frequency (proportion)	Cluster center (CREA *z*, RISC *z*)	Core trait description	PA mean	NA mean	Emotional experience features
High Creativity-High Resilience Synergistic Balance Group (Cluster 2)	350 (38.3%)	(+0.97, +0.50)	Creativity level dominant, strong psychological adaptability	36.78	25.28	Optimal mood: High PA, Low NA
Low Creativity-Low Resilience Burnout Sensitive Group (Cluster 1)	284 (31.0%)	(−0.70, −1.13)	Dual resource reserves low, susceptible to pressure	30.40	28.27	Worst mood: Low PA, High NA
Low Creativity-High Resilience Steady Conservative Group (Cluster 0)	281 (30.7%)	(−0.50, +0.52)	Creative willingness below average, resilience above baseline	33.85	25.47	Balanced mood: Medium PA, Low NA
One-way ANOVA Inter-group F Test				100.16***	21.99***	Extremely significant inter-group mean differences

Clustering indicators are *Z*-standardized scores, diagonal scatter distribution refers to center values; ***p < 0.001.

**Figure 6:**
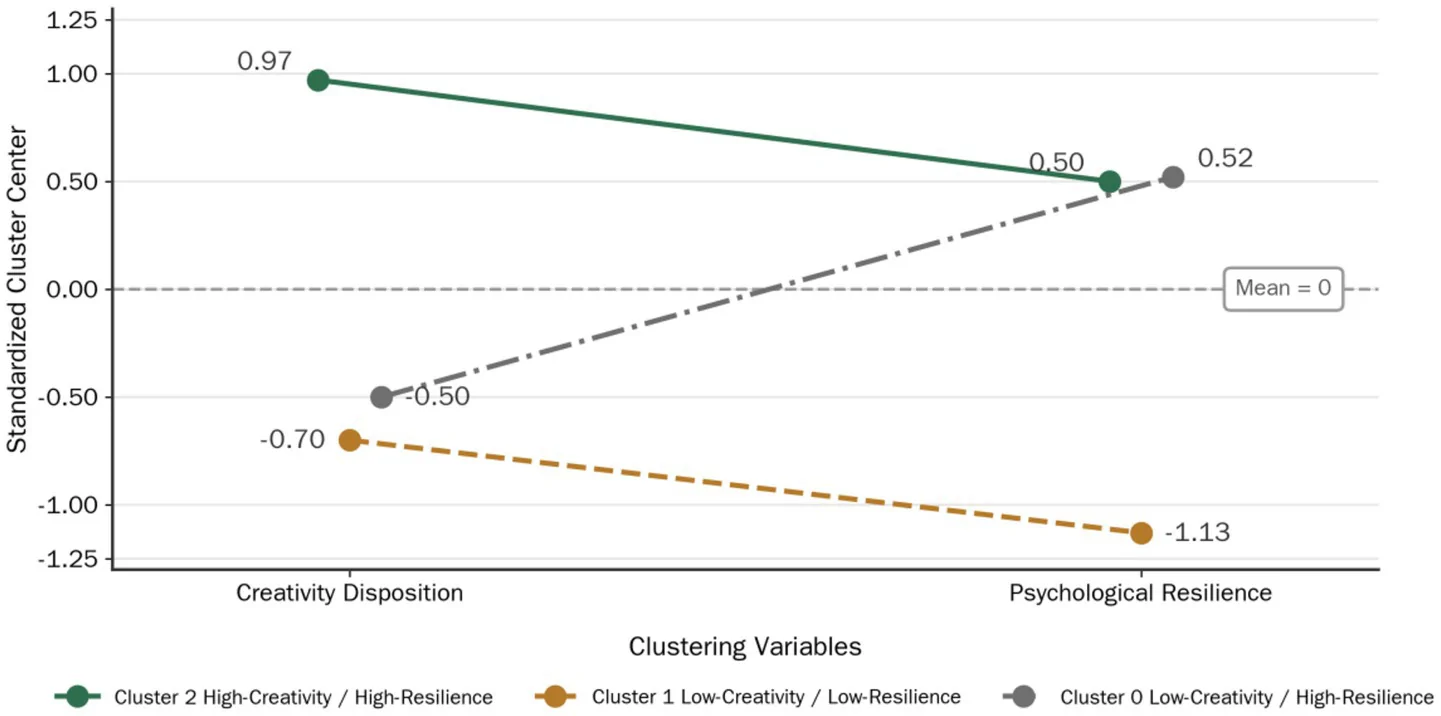
Creator and performer cluster center profile chart based on creativity disposition and psychological resilience. The horizontal axis represents clustering variables, and the vertical axis represents *Z*-standardized cluster centers. The dashed line indicates a sample standardized mean of 0. Values in the figure are the standardized center values for each cluster on creativity disposition and psychological resilience. Cluster 2 represents the High Creativity-High Resilience Synergistic Balance Group, Cluster 1 represents the Low Creativity-Low Resilience Burnout Sensitive Group, and Cluster 0 represents the Low Creativity-High Resilience Steady Conservative Group.

To more intuitively present the standardized center differences of the three groups on the two clustering variables of creativity disposition and psychological resilience, this study mapped a cluster center profile chart (see Figure 6).

Table 10 and Figure 6 collectively show that the three types of creators and performers exhibit relatively clear differentiated profiles in psychological resource allocation.

High Creativity-High Resilience Synergistic Balance Group (Cluster 2, accounting for 38.3% of the sample): This group is higher than the sample standardized mean in both dimensions of creativity disposition and psychological resilience, manifesting as a stronger creative exploration disposition and better psychological adaptability. Regarding emotional states, this group has the highest positive affect (*M* = 36.78) and the lowest negative affect (*M* = 25.28), presenting relatively optimal emotional adaptation characteristics.

Low Creativity-Low Resilience Burnout Sensitive Group (Cluster 1, accounting for 31.0% of the sample): This group is lower than the sample standardized mean in both dimensions of creativity disposition and psychological resilience, especially showing obviously low levels of psychological resilience (*z* = −1.13). Correspondingly, this group has the lowest positive affect (*M* = 30.40) and the highest negative affect (*M* = 28.27), suggesting that they may be more prone to emotional burnout during creative/performance pressure and task challenges.

Low Creativity-High Resilience Steady Conservative Group (Cluster 0, accounting for 30.7% of the sample): This group’s creativity disposition is lower than the sample mean, but psychological resilience is higher than the sample mean (*z* = 0.52). Its positive affect is at an average level (*M* = 33.85), and negative affect is low (*M* = 25.47), indicating that higher psychological resilience may buffer the emotional fluctuations accompanied by lower creative disposition to a certain extent.

## 5 Discussion

### 5.1 Theoretical interpretation of main findings

Through empirical investigation, this study examined the distribution characteristics of core psychological variables among contemporary dance creators and performers of ICH and traditional folk elements. The results show that both the psychological resilience and the total creativity disposition scale scores of this group are above theoretical midpoints, and positive affect is significantly higher than negative affect. This is consistent with related discussions on the distribution of positive psychological resources among performing arts practitioners in existing literature (Hanna, 2014; Perkins and Williamon, 2014).

Theoretically, while acknowledging that scores above a theoretical midpoint only represent descriptive relative levels rather than normative psychological advantages, uncovering these comparatively higher scores implies that participating in ICH-related dance is a highly active psychological coping process rather than mere mechanical execution. After controlling for demographic variables, the concurrent manifestation of positive and negative emotions at mean levels supports the orthogonal theoretical perspective that positive and negative affects belong to two relatively independent systems (Watson et al., 1988). This concurrent feature reflects the dual reality of the ICH contemporary dance creator and performer group: on one hand, translating excellent traditional cultural resources and engaging in artistic exploration often co-occurs with positive emotions such as pride, a sense of value, and feeling inspired; on the other hand, attempting to reconstruct traditional vocabularies in contemporary contexts, developing physical intensity, and the constraints of rehearsal standards are also prone to covary with negative emotions (such as creation/performance pressure). This demonstrates that high emotional significance and high occupational load are coexisting features of the mood states of ICH creative individuals.

### 5.2 Theoretical implications of correlations and statistical indirect pathways

Pathway analysis indicated a significant statistical indirect association of psychological resilience linking creativity disposition and negative affect. It is worth noting that creativity disposition and negative affect originally only had a weak negative correlation (*r* = −0.110) which accounts for roughly 1% of the shared variance (*R*^2^ ≈ 0.012). After controlling for psychological resilience, the direct link between them was no longer significant. This suggests that creativity disposition itself is not directly associated with lower negative affect; its association with lower negative affect is statistically transmitted through the higher psychological resilience levels typically accompanying high-creativity individuals. The openness, desire to explore, and cognitive restructuring flexibility possessed by individuals with high creativity disposition help them build richer psychological resource reserves, serving as an important resource associated with lower emotional burnout related to occupational pressure (Yang et al., 2024; Feng and Wang, 2025).

The increment in explanatory power in the hierarchical regression model verified this: after controlling for demographic backgrounds and creation/performance difficulty, the variance explanation increment of psychological resilience on negative affect is more than five times that of creativity disposition. This suggests that compared to creativity disposition which emphasizes cognitive-volitional attributes, psychological resilience itself, which emphasizes defense and recovery, is the most robust statistical correlate in this study’s framework that is most strongly associated with emotional fluctuations in this artistic group. Considering that more than half of the sample in this study are university students majoring in dance, this finding also echoes conclusions in the field of educational psychology, namely that resilience (such as academic resilience) is a key resource for young student groups that exhibits strong positive links to self-efficacy and emotional stability when facing high-pressure tasks (Yang et al., 2024).

By elucidating this indirect pathway, the present study theoretically expands contemporary performance psychology—shifting the analytic lens from a traditional stress-pathology perspective to a resource-building paradigm within cultural heritage contexts. While the mediation arithmetic holds statistically, building a dominant practical intervention target solely upon this relatively weak baseline would be disproportionate to the data. Instead, this study more specifically affirms that innate creative drive is insufficient to independently buffer severe emotional loads; rather, psychological resilience may serve as a necessary translational capability that converts artistic creativity into sustainable affective well-being.

### 5.3 Discussion on creation/performance pressure, multiple roles

This study shows that there are significant differences in psychological resilience reserves among groups with different subjective perceptions of creation/performance difficulty and pressure. The extremely high-pressure creator and performer group is accompanied by an obvious lack of psychological resilience; moreover, after eliminating the information loss caused by artificial grouping, the continuous gradual attribute of the data also revealed that as the subjective feeling of creation/performance difficulty mounts, the practitioners’ psychological resilience exhibits a significant negative monotonic association. This covariation trend reminds us that contemporary dance creation and performance containing ICH elements often have higher artistic and practical thresholds, and when individuals cross these aesthetic transformation obstacles, their callable psychological resilience reserves are often found to be at lower levels.

In the exploratory analysis of differences in the number of roles, it was found that the mean levels of negative affect for dance creators and performers holding 1–4 roles concurrently remained within a relatively robust fluctuation range. This indicates that due to long-term professional training, dance practitioners exhibit a certain degree of role elasticity toward conventional multiple task burdens (Agres and Chen, 2025).

However, the mean negative affect of the extremely small number of individuals holding 5 roles concurrently in the data presented an exploratory descriptive elevation. Although inter-group significance could not be established in inferential statistics due to the sub-sample size being too small, it still suggests that extreme role configurations may be related to stronger emotional fluctuations. However, given the lack of inferential evidence, this remains a purely exploratory descriptive observation. Future large-scale structural investigations are required to rigorously verify this phenomenon before projecting macro team-management prescriptions.

### 5.4 Research limitations and future prospects

This study provides data references for effectively examining the association between positive traits and emotions of dance creators and performers, but there are several methodological limitations:

First, the limitations of cross-sectional survey design. This study is based on calculations from single time point data; all indirect configurations and regression directions are restricted to descriptive discussions at the statistical association level (Hair et al., 2010) and cannot confirm the causal sequence among creativity disposition, psychological resilience, and emotional states. For instance, while this study hypothesized creativity disposition as the starting point, an equally plausible alternative ordering—consistent with the broaden-and-build theory—is that individuals inherently possessing higher positive affect and psychological resilience gradually develop more exploratory and creative traits over time. Since temporal dynamics cannot be captured through one-time measurements, future research would benefit from multi-timepoint lagged designs that track the longitudinal fluctuations of practitioners’ creative impulses, resilience depletion, and rehearsal-period stresses.

Second, sample skew limits external validity. The sample mainly consists of university teachers and students, and the convenience and snowball sampling methods adopted lead to insufficient representation of other circles such as professional folk artists and senior principal dancers from state-owned and private troupes. As a result, the current psychological profiles may partially reflect the characteristics of academically affiliated practitioners and do not necessarily account for the experiences of seasoned master folk inheritors or veteran actors. Future research could introduce nested multilevel linear models to control for institutional background attributes (e.g., public troupes vs. commercial performance groups) and environmental characteristics such as the types of funding received.

Third, there are measurement precision limitations in the creativity sub-dimension scales. Specifically, while the adaptation process utilized expert peer review to establish face validity and a preliminary readability check, it did not include a large-scale quantitative pilot test to psychometrically calibrate the sub-dimensions prior to formal distribution. Consequently, the low reliability coefficients of each sub-dimension directly restricted the feasibility of conducting inferential path analysis on sub-dimensions. Furthermore, the average variance extracted of the total creativity disposition scale was slightly insufficient, indicating that when the adapted scale involves specific Chinese ICH dance creation, there is still a necessity to refine and expand the items for construct congruence and local equivalence.

Fourth, the potential impact of self-report questionnaire bias. Although tests were within normal ranges, subjective evaluations of respondents’ emotions cannot entirely rule out situational biases brought about by physical and mental fatigue on the rehearsal day or social desirability response tendencies. Additionally, while Harman’s single-factor test was employed as an initial diagnostic, future studies should consider utilizing more robust procedural remedies or marker variable techniques to thoroughly address common method variance.

## 6 Conclusion

Taking 915 contemporary dance creators and performers engaged in works featuring ICH and traditional folk elements as subjects, this study examined the association patterns among their creativity disposition, psychological resilience, and emotional states. Ultimately, this study illuminated the complex psychological orchestration behind cultural transmission, drawing the following conclusions:

(1) The creator and performer group as a whole reports relatively favorable self-assessed levels of creativity disposition and psychological resilience relative to theoretical midpoints, and their mood states are dominated by positive affect; however, it cannot be ignored that about one-third of the practitioners are facing high levels of subjective creation/performance difficulty and pressure.

(2) Psychological resilience presents a significant indirect association between creativity disposition and negative affect within the hypothesized model framework. The negative association between creativity disposition and negative emotion is not direct, but is instead indirectly explained through its shared variance with psychological resilience. This suggests that incorporating psychological resilience into support mechanisms may serve as a beneficial supplementary approach to addressing practitioners’ occupational emotional load (Zhang et al., 2025; Li H. et al., 2025), as bolstering practitioners’ adaptive recovery capabilities may effectively bridge the psychological gap between ambitious creative expectations and reality-induced performance anxiety.

(3) Based on traits, the creator and performer group can be clustered into three main subtypes: “High Creativity-High Resilience Synergistic Balance,” “Low Creativity-High Resilience Steady Conservative,” and “Low Creativity-Low Resilience Burnout Sensitive.” Among them, the “Burnout Sensitive” type lacking dual resources suffers the greatest negative emotional distress.

## Data Availability

The original contributions presented in the study are included in the article/Supplementary material, further inquiries can be directed to the corresponding author.
