# Supplementary material for: Associations among creativity disposition, psychological resilience, and emotional states in contemporary dance practitioners of intangible cultural heritage
Source: Front Psychol. 2026 Aug 21;17:1909664. doi: 10.3389/fpsyg.2026.1909664 (PMC13541435; doi:10.3389/fpsyg.2026.1909664)
Supplement: Supplementary file 1 [file Data_Sheet_2.DOCX]

**Part 1: Survey Questionnaire**

**Questionnaire on Artistic Creation and Psychological Characteristics of Dance Creators and Performers**

[Introduction and Informed Consent]

Dear Dance Practitioners/Learners:

Hello! We are an academic team conducting interdisciplinary research in art and psychology. We are currently conducting an empirical study on the artistic creation traits and psychological states of dance creators and performers in specific cultural contexts (such as the contemporary transformation of intangible cultural heritage).

This questionnaire is completely anonymous. All collected data will only be used for academic statistics and research report writing, and your personal privacy will absolutely not be disclosed. Your participation is entirely voluntary. If you feel any discomfort during the answering process, you can terminate and exit at any time.

It will take about 5-8 minutes to complete the questionnaire. Your truthful answers are of great academic value to our research.

**1. Have you read the above information and voluntarily agree to participate in this survey? [Single Choice]**

A. Yes, I agree to participate (Continue)

B. No, I do not agree (End questionnaire)

**[Part I: Basic Information]**

**2. [Screening Question] Have you participated in the creation, rehearsal, or performance of contemporary dance or dance drama works containing “Intangible Cultural Heritage (ICH)” or “traditional folk elements”? [Single Choice]**

A. Yes, I have participated (Continue)

B. No, I have not participated (End questionnaire, thank you for your participation)

**3. Your gender: [Single Choice]**

A. Male

B. Female

C. Prefer not to say

**4. Your age group: [Single Choice]**

A. 18-22 years old

B. 23-28 years old

C. 29-35 years old

D. 36 years old and above

**5. Your main occupational identity or professional status is: [Single Choice]**

A. Employed staff in professional troupes (dance companies, song and dance theaters, etc.)

B. Dance faculty at art institutions/comprehensive universities

C. Dance student at art institutions/comprehensive universities

D. Independent choreographer / Freelance dancer

E. Other: _________

**6. What is the main role you play in dance works containing ICH elements? (Multiple choices allowed) [Multiple Choice]**

A. Chief choreographer / Co-choreographer

B. Principal dancer (Lead role/Lead dancer)

C. Ensemble dancer

D. Rehearsal director / Teacher

E. Other: _________

**7. Subjectively, how difficult and psychologically stressful do you think it is to carry out the “contemporary dance transformation” of “traditional elements” in creation/performance? [Single Choice]**

1 point (No difficulty/pressure at all) / 2 points / 3 points (Moderate pressure) / 4 points / 5 points (Extreme difficulty/Extreme pressure)

**[Part II: Psychological and Creative Traits Experience]**

**Module A**

**Instructions: The following describes states when people face challenges. Please choose based on your true feelings within the past month. (1=Never, 2=Rarely, 3=Sometimes, 4=Often, 5=Almost always)**

8. I am able to adapt to change.

9. I can handle unexpected emergencies or difficulties.

10. When facing challenges or difficulties, I tend to see the positive side of things.

11. No matter what pressure I face, I can stay focused on my work or rehearsal.

12. I can recover quickly after experiencing setbacks, illness, or injury.

13. Even when encountering great resistance, I will stay strong to achieve my artistic goals.

14. I feel that no matter what happens, I can mostly handle it with ease.

15. Past successes in overcoming difficulties give me confidence to face new challenges.

16. I do not give up easily when facing tricky or unsatisfactory situations.

17. When problems arise, I believe I can usually find a solution.

**Module B**

**Instructions: The following are about your habits in dance art creation/performance. Please choose based on your first instinct. (1=Completely disagree, 2=Somewhat disagree, 3=Neutral/Uncertain, 4=Somewhat agree, 5=Completely agree)**

18. I like to try non-traditional and even risky new movements in rehearsals.

19. Faced with complex cultural adaptation tasks, I usually find them interesting rather than intimidating.

20. I often have a strong desire to learn about the historical stories and folk customs behind specific dances.

21. When hearing a specific rhythm or melody, unexpected images often come to my mind.

22. I don’t mind making a fool of myself during creation, because that is often the beginning of inspiring new ideas.

23. I like to take on abstract themes that others consider “difficult to express through dance.”

24. When seeing an unencountered local art, I immediately want to analyze how it was formed.

25. It is relatively easy for me to translate abstract concepts into concrete body language.

26. In pursuit of better stage effects, I am willing to break conventional rehearsal methods.

27. When encountering creative bottlenecks, I can usually quickly come up with various alternative solutions.

28. I often question existing classic choreography routines and try to find another possibility.

29. I am never satisfied with just dancing simple works that I am highly familiar with.

**Module C**

**Instructions: Please recall the frequency with which you experienced the following emotions in the past two weeks (especially participating in dance creation and performance). (1=Very slightly or not at all, 2=A little, 3=Moderately, 4=Quite a bit, 5=Extremely)**

30. Interested

31. Upset

32. Excited

33. Nervous

34. Strong

35. Guilty

36. Inspired

37. Scared

38. Enthusiastic

39. Jittery

40. Proud

41. Irritable

42. Alert

43. Ashamed

44. Attentive

45. Distressed and sad

46. Active

47. Hostile

48. Determined

49. Afraid

**[Conclusion and Psychological Support Guidelines]**

This is the end of the questionnaire. Thank you again for your participation!

The emotional states measured in this study are for group statistics only and do not represent a clinical diagnosis. If you have recently been bothered by persistent emotional distress that is difficult to regulate, it is recommended that you seek help from the mental health center of your institution or call the national general psychological intervention hotline (e.g., 400-161-9995).

**Part 2: Codebook and Scoring Rules**

**I. Basic and Demographic Variables (Demographics)**

| Variable | Label | Type | Values & Coding |
| --- | --- | --- | --- |
| ID | Sample ID | Numeric | 001, 002, 003… |
| Gender | Gender | Nominal | 1=Male, 2=Female, 3=Prefer not to say |
| Age | Age group | Ordinal | 1=18-22 yrs, 2=23-28 yrs, 3=29-35 yrs, 4=36 yrs and above |
| Occu | Occupational identity | Nominal | 1=Troupe actor, 2=University teacher, 3=University student, 4=Independent dancer, 5=Other |
| Role_1 | Role: Choreographer (Multiple choice split) | Dummy | 0=Unselected, 1=Selected |
| Role_2 | Role: Principal (Multiple choice split) | Dummy | 0=Unselected, 1=Selected |
| Role_3 | Role: Ensemble (Multiple choice split) | Dummy | 0=Unselected, 1=Selected |
| Role_4 | Role: Rehearsal director (Multiple choice split) | Dummy | 0=Unselected, 1=Selected |
| Role_5 | Role: Other (Multiple choice split) | Dummy | 0=Unselected, 1=Selected |
| Pres | Subjective creation pressure | Scale | 1 (Extremely small) to 5 (Extremely large) |

**II. Psychological Resilience Scale Scoring (CD-RISC-10)**

Scale variable names: RISC_01 to RISC_10. Measured on a 5-point Likert scale, no reverse-scored items.

Calculation formula for total psychological resilience score:

$\text{RISC}\text{\_}\text{Total}\text{=}\sum_{\text{i}\text{=1}}^{\text{10}} \text{R}\text{ISC}\text{\_}\text{i}$	(1)

**III. Creativity Disposition Test Scoring (Modified Williams Creativity)**

Scale variable names: CREA_01 to CREA_12. Measured on a 5-point Likert scale, no reverse-scored items.

Risk-taking calculation formula:

$\text{CREA}\text{\_}\text{Risk}\text{=}\text{CREA}\text{\_01+}\text{CREA}\text{\_05+}\text{CREA}\text{\_09}$	(2)

Challenge calculation formula:

$\text{CREA}\text{\_}\text{C}\text{h}\text{al}\text{=}\text{CREA}\text{\_02+}\text{CREA}\text{\_06+}\text{CREA}\text{\_12}$	(3)

Curiosity calculation formula:

$\text{CREA}\text{\_}\text{Curi}\text{=}\text{CREA}\text{\_03+}\text{CREA}\text{\_07+}\text{CREA}\text{\_11}$	(4)

Imagination calculation formula:

$\text{CREA}\text{\_}\text{Imag}\text{=}\text{CREA}\text{\_04+}\text{CREA}\text{\_08+}\text{CREA}\text{\_10}$	(5)

Total creativity score calculation formula:

$\text{CREA}\text{\_}\text{Total}\text{=}\sum_{\text{j}\text{=1}}^{\text{12}} \text{C}\text{REA}\text{\_}\text{j}$	(6)

**IV. Emotional States Scale Scoring (PANAS)**

Scale variable names: PANAS_01 to PANAS_20. Measured on a 5-point Likert scale. This scale contains two orthogonal dimensions, which must be scored separately; they absolutely cannot be added together to calculate a total score.

Positive Affect (PA) score calculation formula: (Items: 1,3,5,7,9,11,13,15,17,19)

$\text{PANAS}\text{\_}\text{PA}\text{=}\sum_{\text{k}\text{∈\{1,3,5,7,9,11,13,15,17,19\}}} \text{P}\text{ANAS}\text{\_}\text{k}$	(7)

Negative Affect (NA) score calculation formula: (Items: 2,4,6,8,10,12,14,16,18,20)

$\text{PANAS}\text{\_}\text{NA}\text{=}\sum_{\text{k}\text{∈\{2,4,6,8,10,12,14,16,18,20\}}} \text{P}\text{ANAS}\text{\_}\text{k}$	(8)
